# Acute Acalculous Cholecystitis due to primary acute Epstein-Barr virus infection treated with laparoscopic cholecystectomy; a case report

**DOI:** 10.1016/j.amsu.2018.10.010

**Published:** 2018-10-10

**Authors:** Kamal N. Rezkallah, Khalid Barakat, Abdurraheem Farrah, Shesh Rao, Monica Sharma, Shyam Chalise, Teresita Zdunek

**Affiliations:** a Internal Medicine Department, St. Joseph Hospital, 2900 N Lake Shore Dr, Chicago, IL, 60657, USA; b Clinical Pathology Department, St. Joseph Hospital, 2900 N Lake Shore Dr, Chicago, IL, 60657, USA

**Keywords:** EBV, Epstein-Barr virus, Acalculous, Cholecystitis, Gallbladder, Case report, EBV, Epstein Barr virus, AAC, Acute Acalculous Cholecystitis, SCARE, Surgical Case Report, HIDA, Hepatobiliary iminodiacetic acid

## Abstract

**Introduction:**

Epstein Barr virus (EBV) is a human herpes virus 4, transmitted through intimate contact between susceptible persons and asymptomatic EBV shedders. It usually presents with fever, pharyngitis and lymphadenopathy. Majority of individuals with primary EBV infection recover uneventfully. Acute Acalculous Cholecystitis (AAC) is usually seen in hospitalized and critically ill patients with major trauma, shock, severe sepsis, total parenteral nutrition and mechanical ventilation.

**Case presentation:**

We report a 25-year- old woman presented with acute Epstein-Barr Virus (EBV)infection and hepatobiliary iminodiacetic acid (HIDA) scan confirmed presence of Acute Acalculous Cholecystitis (AAC). Conservative management was advised initially, but she had a laparoscopic cholecystectomy due to intolerable abdominal pain.

**Conclusion:**

AAC is a rare complication of acute EBV infection and it is usually managed conservatively, although our patient had laparoscopic cholecystectomy due to intolerable abdominal pain.

## Highlights

• Acute Acalculous Cholecystitis (AAC) is a rare complication of acute EBV infection.
• AAC should be suspected in patient with acute EBV infection, presenting with abdominal pain.
• Management is usually conservative without surgical intervention.

## 1 Introduction

Epstein-Barr Virus (EBV) is a human herpes virus 4, transmitted through intimate contact between susceptible persons and asymptomatic EBV shedders. It usually presents with fever, pharyngitis and lymphadenopathy [1]. Majority of individuals with primary EBV infection recover uneventfully [2]. Acute Acalculous Cholecystitis (AAC) is usually seen in hospitalized and critically ill patients with major trauma, shock, severe sepsis, total parenteral nutrition and mechanical ventilation [3,4]. This work has been reported in line with the surgical case report (SCARE) criteria [5].

## 2 Case presentation

We report a 25-year- old woman who presented with fever T 38.6C, sore throat, abdominal pain, nausea, vomiting and anorexia. On physical exam, she had right upper quadrant abdominal tenderness without signs of lymphadenopathy. She was in her usual state of health as an average healthy woman with medical history of polycystic ovarian syndrome, gastroesophageal reflux disease and mild intermittent asthma until ten days prior to admission when she had headache, fever, cough and muscle aches. Her primary care physician prescribed her a prophylactic course of Oseltamivir 75 mg twice daily for five days and after the third day of treatment, her symptoms continued to worsen with development of abdominal pain and she walked into our emergency room. Initial investigations showed: negative Flu/RSV by PCR, WBC count (6.3 k/mm cu; reference range 4.0–11.0 k/mm cu), lymphocytes count (30%; reference range 15%–50%), atypical lymphocytes (24%, 1.5 k/mm cu; reference range 0%, 0 to 0.1 k/mm cu), conjugated bilirubin (0.5 mg/dL; reference range 0.0–1.0 mg/dL), alanine aminotransferase (116 U/L; reference range 7–52 U/L), aspartate aminotransferase (94 U/L; reference range 13–39 U/L), alkaline phosphatase (154 U/L; reference range 35–104 U/L), positive EBV VCA IgM antibody, negative EBV VCA IgG antibody and high EBV count by PCR (115,000 cpy/mL, 5.1 log cpy/mL; reference range less than 390 cpy/mL, less than 2.6 log cpy/mL) which confirmed presence of acute EBV infection.

**Fig. 1:**
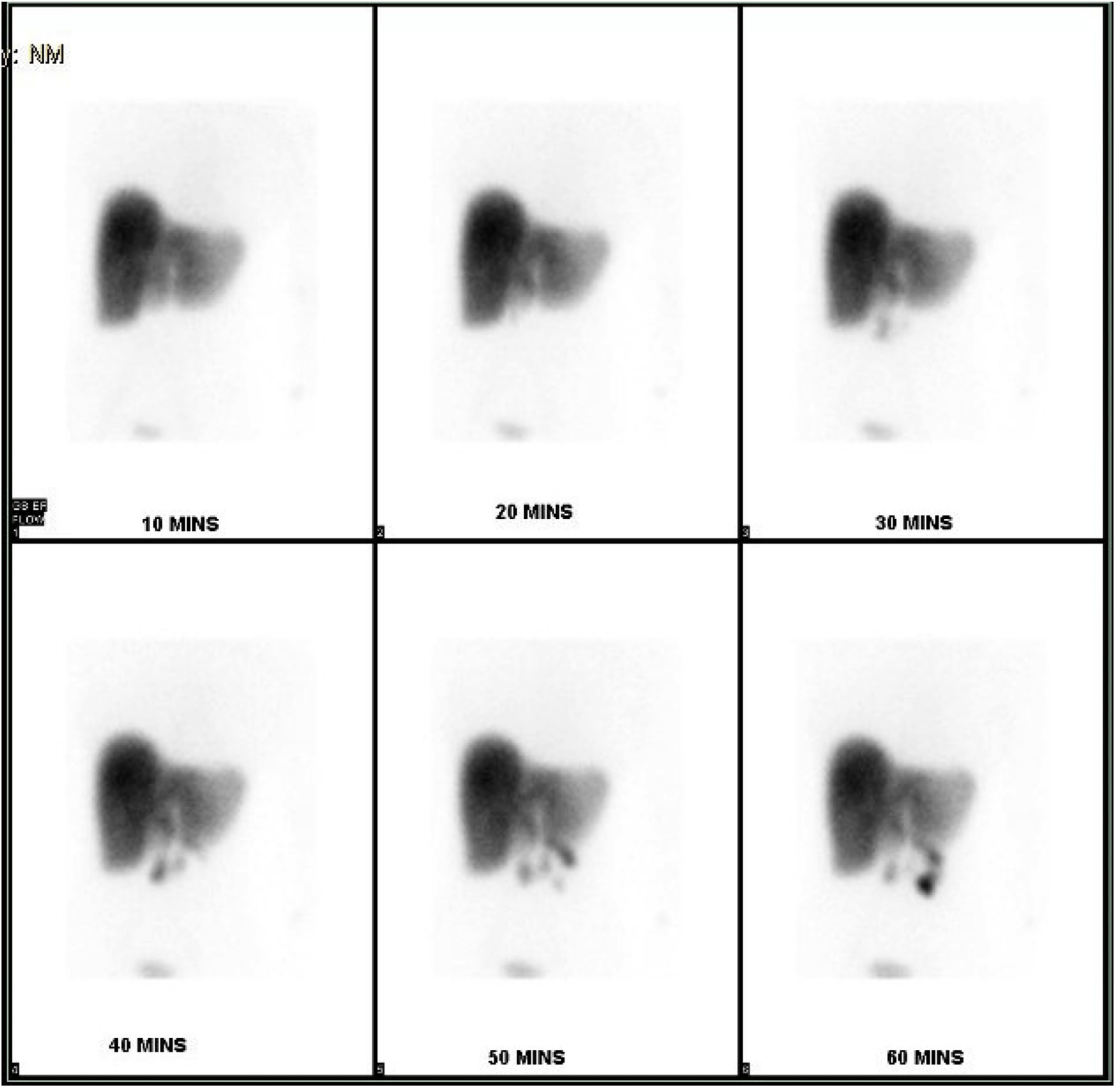
HIDA scan showing non-accumulation of the isotope within the gallbladder.

On day 2 of admission, increasing intensity of abdominal pain with worsening of liver function, warranted further investigations: conjugated bilirubin (1.4 mg/dL; reference range, 0.0–1.0 mg/dL), alanine aminotransferase (127 U/L; reference range 7–52 U/L), aspartate aminotransferase (129 U/L; reference range 13–39 U/L) and alkaline phosphatase (154 U/L; reference range 35–104 U/L). Abdominal ultrasonography was unremarkable and abdominal computed tomography showed mild gallbladder distension, mild gallbladder wall thickening (2.5 mm) and mild pericholecystic fluid collection with no layering stones, sludge or biliary ductal dilation. Hepatobiliary iminodiacetic acid (HIDA) scan showed non-accumulation of the isotope within the gallbladder which confirmed the presence of AAC [6], [Fig. 1].

On day 4 of admission, abdominal pain was worsening and her blood pressure dropped into 80/50 mm/Hg. Conservative management was advised initially, but her abdominal pain was intolerable so she opted for a laparoscopic cholecystectomy. Intraoperatively, the gall bladder was found to be edematous, markedly inflamed and no gallstones were found. The final pathology report of the removed gallbladder showed AAC. She was given a single dose of 2 gm of intravenous Cefazolin preoperatively. She was not given glucocorticoids or acyclovir. Her symptoms improved after surgery and she was discharged on the fifth day of admission. Liver function returned to normal levels two weeks after surgery.

## 3 Discussion

Although majority of cases with primary acute EBV infection recover without sequelae, few cases of AAC have been reported as a complication of primary acute EBV infection [7]. Kottanattu et al. reported in a systemic review of the literature, 37 cases of AAC in primary acute EBV infection which were published between 1966 and 2016, all cases always recovered without surgery or corticosteroids, following a hospital stay of 25 days or less [8]. Agergaard and Larsen did another literature review showed 26 cases of AAC in acute primary EBV infection, only one patient had laparoscopic cholecystectomy [9] and the rest recovered without surgery, also broad-spectrum antibiotics have no impact on severity of disease's symptoms, course or length of hospital stay [10]. The distinguishing feature of our patient that she looked seriously sick, on and throughout admission. Her abdominal pain was worsening, she did not tolerate pain medications and she had high EBV viral load which usually correlates with disease severity [11]. Conservative management was advised initially but she opted for surgery and had laparoscopic cholecystectomy [12,13]. She received a single dose of intravenous Cefazolin preoperatively. She was discharged on day 5 of admission.

## 4 Conclusion

AAC is a rare complication of primary acute EBV infection which is usually managed conservatively without surgery, however, our patient had laparoscopic cholecystectomy due to intolerable abdominal pain. AAC should be suspected in patients with acute EBV infection, presenting with abdominal pain.

## Ethical approval

Our institution does not require ethical approval for publishing case report.

## Sources of funding

This research did not receive any specific grant from funding agencies in the public, commercial, or not-for-profit sectors.

## Author contribution

First Author: Kamal Rezkallah.

Corresponding Author: Kamal Rezkallah.

Kamal Rezkallah: Literature review, data collection, original draft writing and final approval of the draft to be submitted.

Khlid Barakat, Abdurraheem Farrah, Shesh Rao, Monica Sharma, Shyam Chalise and Teresita Zdunek: Literature review, draft revising and final approval of the draft to be submitted.

## Conflicts of interest

None.

## Research registration number

None.

## Guarantor

Kamal Rezkallah.

## Provenance and peer review

Not commissioned, externally peer reviewed.

## Reproduction

Black and White.

## Consent

Written informed consent was obtained from the patient for publication of this case report and accompanying images. A copy of the written consent is available for review by the Editor-in- Chief of this journal on request.

## Appendix A Supplementary data

SCARE Guideline Checklist.docx SCARE Guideline Checklist.docx
